# Magnitude of NNM and associated factors among Newborns delivered at the North Shewa zone Public Health Hospital, Central Ethiopia: A multi-level analysis

**DOI:** 10.3389/fpubh.2022.979636

**Published:** 2022-11-07

**Authors:** Girma Garedew Goyomsa, Birhanu Senbeta Deriba, Meseret Moroda Wadejo, Sisay Abebe Debela, Abebe Feyissa Amhare

**Affiliations:** Department of Public Health, College of Health Sciences, Salale University, Fitche, Ethiopia

**Keywords:** magnitude, neonatal near miss, associated factor, North Shewa zone, Ethiopia

## Abstract

**Background:**

Neonatal near miss refers to a condition where a newborn is close to death within the first 28 days of life but ultimately survives either by chance or because of the quality of care they received. It is considered a major public health problem that contributes to the global burden of disease in less developing countries. For every death due to NMM, many others develop a severe complication. Despite this grim reality, there seems to be a gap in terms of the magnitude of and predictors of NNM in Ethiopia, where the previous study focused on neonatal death investigation. This study aimed to determine the magnitude of NNM and its determinants among the neonates delivered in the North Shewa zone, Central Ethiopia.

**Methods:**

A facility-based cross-sectional study was conducted using a systematic random sampling technique among 747 newly delivered babies in the North Shewa zone public hospital from January 30 to June 30, 2021. Neonatal near misses were identified with the help of the World Health Organization labeling criteria. Collected data were coded, entered, and cleaned by using Epi data 4.4.6 and analyzed using SPSS software (version 26) for analysis. Descriptive statistics were used to compute summary statistics and proportions. Variables at a cutoff value of 0.25 on bivariate and 0.05 on multivariate logistic regression were used to identify predictors.

**Result:**

The prevalence of NNM was 35.3% (95% CI = 31.9–38.6) per 1,000 live births. Participant occupation [AOR: 0.55, CI: 0.33–0.90], marital status [AOR: 2.19; CI: 1.06–4.51], instrumental delivery [AOR: 1.98; CI: 1.10–3.55], intrapartum hemorrhage [AOR: 2.27; CI: 1.03–5.01], abortion history [AOR: 1.59; CI: 1.03–2.44], mal-presentation [AOR: 1.77; CI: 1.14–2.77], premature rupture of membrane [AOR: 2.36; CI: 1.59–3.51], and pregnancy-related infection [AOR: 1.99; CI: 1.14–3.46] were found to have statistically significant association.

**Conclusion and recommendation:**

One-third of neonates face serious neonatal health conditions. Given this, addressing modifiable obstetric risk factors through providing skilled and quality care to mothers during pregnancy and during and after childbirth was important for improving neonatal health. Additionally, strengthening antenatal care services to minimize the infection occurring during pregnancy through the provision of appropriate services and counseling about the consequences of abortion was essential in reversing the problem.

## Background

The neonatal phase, which lasts from birth to 28 days of age, is when a child is the most vulnerable. Children are at the most risk of death during the first month of life (1, 2). Using a near-miss approach in neonatal health is a novel concept for inventing tools to improve the quality of perinatal care (3). An NNM is a situation where neonates suffer severe complications during the first month of life and come nearly close to death but survive, either by chance or because of the good quality of care they receive (4).

To date, there is no universally accepted criterion for identifying the near-miss case in neonates, as many researchers used several scoring mechanisms. While some define NNM as a situation where newborns suffer a life-threatening condition following birth but survive the first 28 days of life, others define it as a shorter life span of seven days. There is also disagreement about the most appropriate markers of illness severity indicative of a near miss. The lack of consistent criteria used in categorizing and defining near-miss challenges every country's response to reducing near misses. Currently, near-miss cases were identified using three criteria: clinical-based, laboratory-based, and management-based criteria (4, 5).

NNM occurs more frequently than neonatal death, i.e., for every one death, many others develop severe complications. In 2019, around 2.4 million deaths occurred in children globally. Prematurity, asphyxia, low birth weight, and infection account for ~80% of neonatal illness and mortality. Approximately 75% of these deaths occur during the first week of life, with 25-45% occurring within the first 24 h (6, 7). This risk makes the day of their birth the most dangerous day for babies in nearly every country (8). Neonatal health problems are an unaccomplished agenda of the MDG and a continued target of sustainable development goal (SDG) by 2030, which calls for an end to preventable infant and child deaths with all countries aiming to reduce neonatal mortality to <12 deaths per 1,000 births (9, 10). Even though the world gained significant achievement in child survival, the WHO Regional Office for Africa has shown slow progress in the reduction of neonatal death (52 per 1,000 live births), which is over seven times higher than the WHO Regional Office for Europe (seven per 1,000 live births) (11). Despite the recorded remarkable decline in post-neonatal mortality in Ethiopia, neonatal mortality remained very high, i.e., about 63% (29/1,000 live births) of infant deaths occurred in the first month of life compared to 37% (19/1,000 live births) of the deaths occurring in the remaining 11 months of life (12).

The neonatal problem remains invisible due to a lack of continuity between maternal and child health programs, as the care of the newborn has fallen through the cracks between the care of the mother and the care of the older child (13). High-impact treatments, such as essential newborn care, kangaroo mother care, and health sector development strategies, have been demonstrated to cut neonatal mortality rates by 50% Evidence showed that the implementation of high-impact interventions such as essential newborn care, kangaroo mother care, and health sector development plan has helped to cut neonatal death by 50% (14–16). Despite these efforts, neonatal morbidity and mortality rates remain high (17–19). Thus, the identification of factors contributing to severe neonatal illness along the continuum of care—during pregnancy, delivery, and the postnatal period—is more likely to contribute to the improvement of child health (5, 20, 21).

Despite the seriousness of the problem, only a few studies examined the system to identify how some neonates managed to evade death and their predisposing factors. Therefore, this study aimed to fill this gap in the literature by determining the magnitude and predisposing factors of a neonatal near miss.

## Methods

### Study area and period

**Figure 1:**
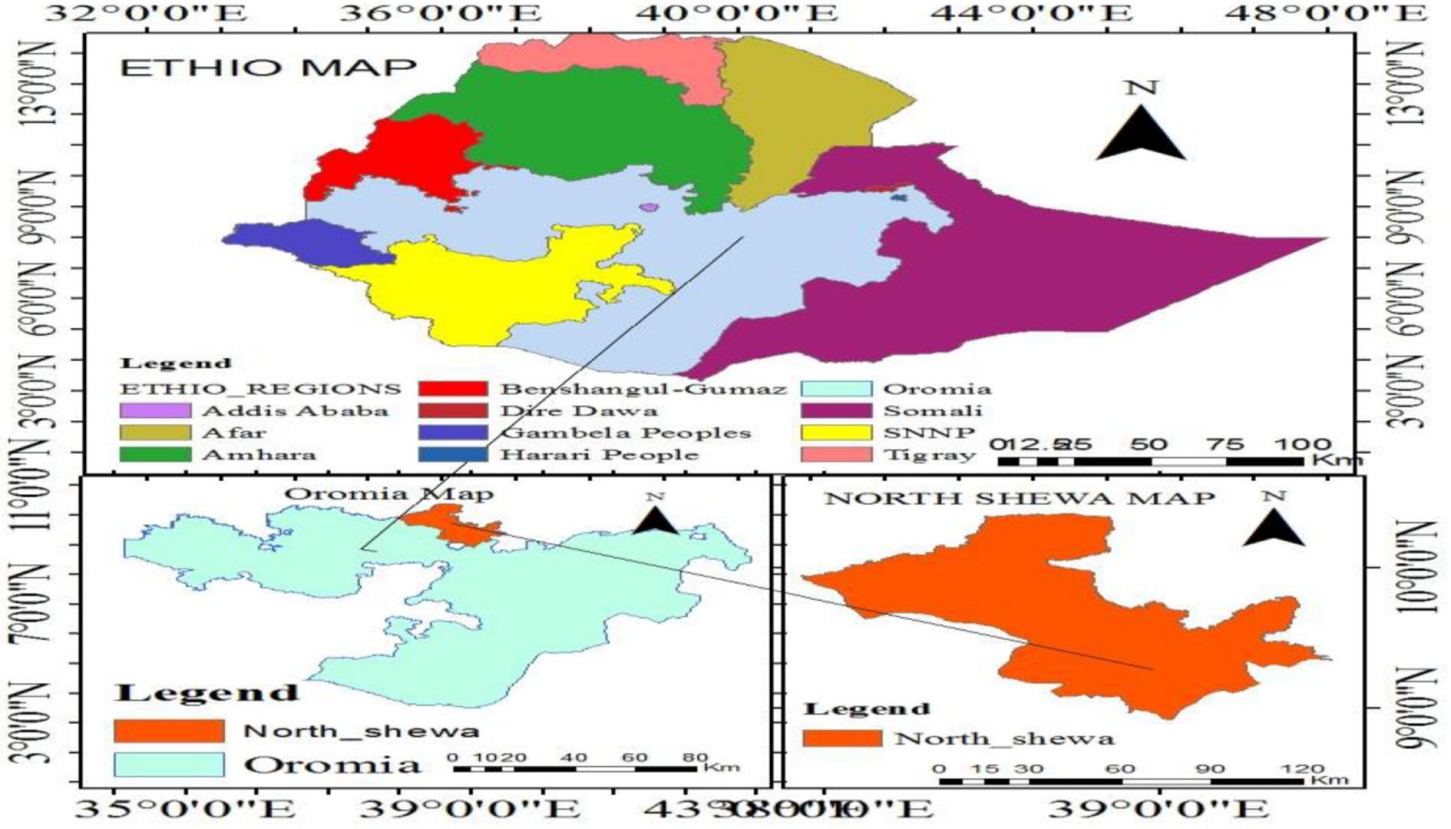
Map of the study area, North Shewa zone, Central Ethiopia, 2021.

The North Shewa zone is located in the Oromia regional state of Ethiopia, 112 km northwest of the capital city, Addis Ababa (Figure 1). The zone has a total population of about 1,639,586, of which 717,552 and 922,034 were men and women, respectively, with a majority of the population (89.75%) residing in rural areas. There are about 68 public health facilities (64 health centers and four public hospitals) and 30 private medium clinics providing service to the community, with only four hospitals (Fiche, Kuyu, Muka Turi, and Dera hospital) providing intensive care services for newborn babies and obstetric or gynecologic related services for mothers. All private clinics did not provide delivery services.

Overall, the selected hospital oversees 7,453 deliveries annually. Fiche general hospital is located in Fiche town, the capital city of the North Shewa zone, and performs more than 2,487 annual deliveries, with ~389 deliveries attended through cesarean section (CS). Kuyu hospital is located in Garba Guracha town, 43 km from Fiche town. It provides services for two adjacent woredas and oversees around 2,004 deliveries annually, of which 289 were conducted with CS. Similarly, Muka Turi hospital and Dera hospital oversee 1,317 and 1,645 deliveries annually, with 225 and 253 deliveries conducted through cesarean sections, respectively. The study was conducted among neonates born to pregnant women admitted to the North Shewa Zone Public Health Hospital from January 30 to June 30, 2021, at four general hospitals.

### Study design and participants

A facility-based quantitative cross-sectional study was conducted among mothers of neonates delivered and admitted to postnatal or neonatal wards in selected hospitals within 28 days of birth. The source population included all newborns delivered at public health hospitals in the North Shewa zone.

### Inclusion and exclusion criteria

All new neonates from their mothers and neonates who were within the first 28 days of life were included in the study, whereas neonates from mothers with ectopic pregnancy and twin delivery were excluded. Additionally, neonates referred from other facilities were also excluded, as it was difficult to identify the pre-referral health condition of the newborn.

### Sample size determination and sampling procedures

**Figure 2:**
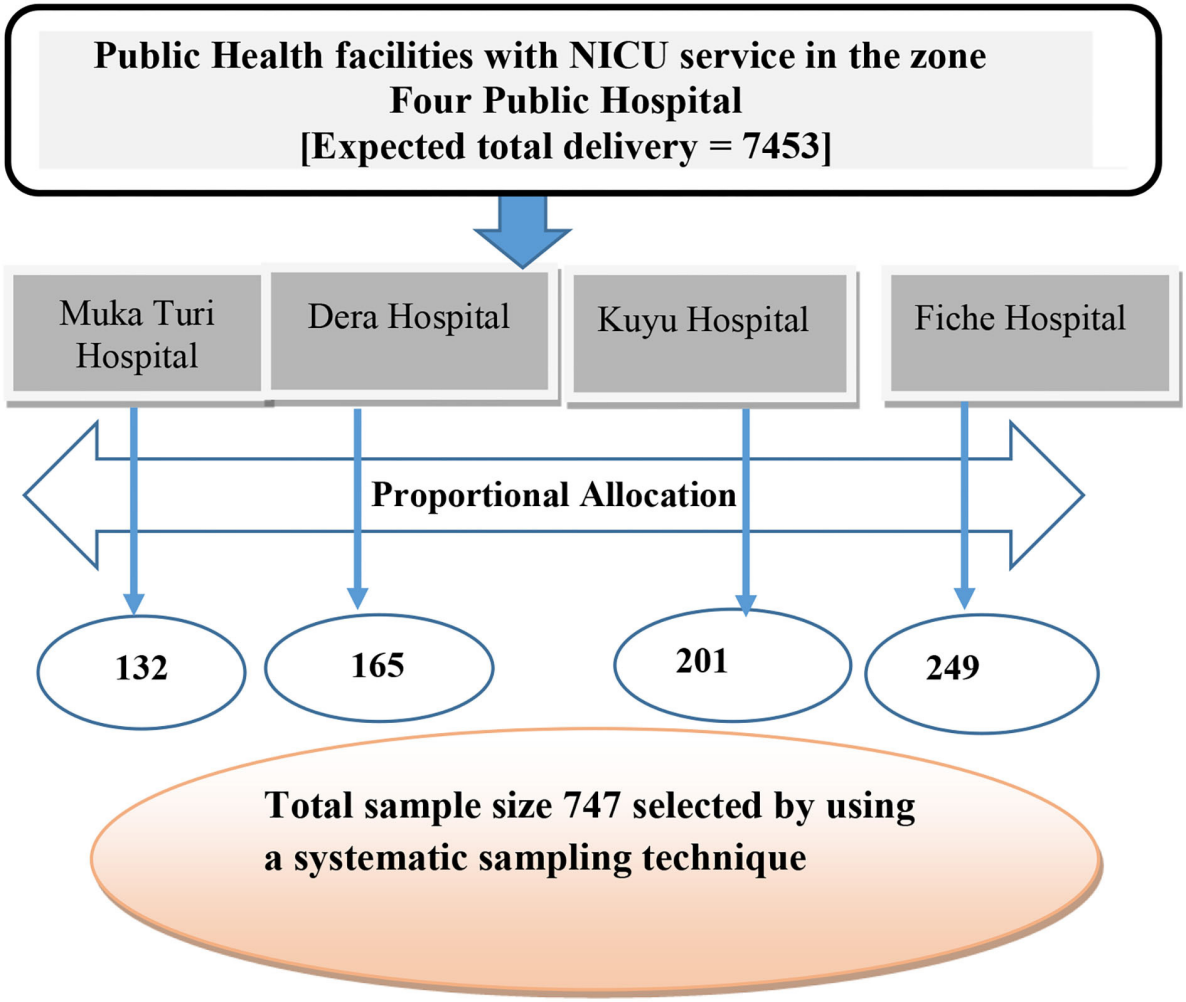
Schematic presentation of sampling technique North Shewa zone public health facilities, Central Ethiopia, 2021.

The required sample size for the study was determined using a single population proportion formula. Forty-five percent of the true population proportion of NNM among newborns from the previous study (21), 95% confidence level, 80% power, and 0.06% estimated precision were considered in the estimation of the required sample size calculation. Accordingly, a total of 679 sample size was determined to be enrolled. The sample size was further inflated by a 10% nonresponse rate, and the final sample size was 747. There are 68 public health facilities (four government hospitals and 64 health centers). All public health facilities providing intensive neonatal care, i.e., four public hospitals, were included in the study. The desired number of participants at each health facility was proportionally allocated by averaging trends of the preceding years' client flow of similar months (i.e., January to June). Individual participant was approach through calculating sampling interval K [N/n, where N is the preceding year average total number of births at the selected public health facilities in the same months and n is the final sample calculated]. Accordingly, of the total number of births [*N* = 7,453], 747 (n) births included in the study yielded a sampling interval of nine. The random starting point was selected by lottery method among the first nine participants from the postnatal registration log book. Figure 2 depicts a schematic presentation of the sampling procedure for the study.

### Data collection personnel, tools, and procedures

The pre-tested structured interviewer-administered questionnaires, along with the review of medical records, were used to collect data from the participant on sociodemographic characteristics, obstetric history, current pregnancy complications, and pregnancy outcomes up to 28 days of life. The questionnaire was initially designed in English and translated into the Afaan Oromo language by a person hired from the zonal cultural and tourism office; the questionnaire was then translated back into English by a third person to check for consistency. The lead investigator oversaw the data collection process that gathered information from the participants over the course of the study's five-month duration. Trained data collectors, four Bachelor of Science (BSc) midwives, and four BSc neonatal nurses collected data in the class arranged for data collection after the neonates were assured of survival. Eight health extension workers were recruited to collect data after the infant was discharged from the hospital for a period of 28 days. The experts who gathered the data were briefed beforehand on the purpose of the study and the procedures for participant selection. Special training was provided for health extension workers on the common criteria used to identify a life-threatening condition in a newborn. All data were collected under the direct supervision of the assigned supervisors.

## Study variables

Study variables were divided into three categories to match the factors associated with an NNM case at different levels. The first category was the proximal factors and included modes of delivery, sex of the newborn, pregnancy, and maternity-related factors. The second included intermediate factors, such as birth interval, pregnancy intention, number of children, ANC utilization, frequency of ANC utilization, and types of pregnancy. The third category of distal factors included major sociodemographic and economic status-related characteristics, such as age, educational status, marital status, and income level (21–24).

### Measurement

The primary outcome variable was NNM, which was classified as present or absent at the end of the follow-up. Outcome variables were identified using three basic criteria: clinical-based, laboratory-based, and management-based criteria.

*Clinical-based criteria* refer to an APGAR score of <7 at the fifth minute, absence of regular breathing, cardiac arrest, persistent bradycardia <80 beats per minute, visible jaundice in first 24 h of delivery, persistent tachycardia >200 beats per minute, birth weight <1,500 g, gestational age < 33 completed weeks (collectively known as pragmatic criteria), seizures, inability to suck within 12 h or inability to suck with six attempts without pause, apathy, poor tolerance to feeding within 12 h, abdominal distension, vomiting, and anuria >24 h. *Laboratory-based criteria* refer to any active non-traumatic bleeding, hematuria, septicemia, and encephalopathy. *Management-based criteria* refer to any intubation, cardiopulmonary resuscitation, use of vasoactive drugs, blood transfusion (simple blood transfusion and exchange blood transfusion), use of anticonvulsants, phototherapy, and parenteral antibiotics (3–5).

Health professionals (midwife, neonatologist, and nurse) began evaluating the newborns as soon as they arrived in the hospital, typically during the early neonatal period. A health extension worker evaluated the newborn's health from the time of release from the hospital through the first 28 days of life. There were follow-ups after the hospital discharge until the 28th day. Near-miss cases were reported when a newborn met at least one of the criteria for the disease, including severe morbidity/clinical criteria, pragmatic criteria, laboratory criteria, or management requirements, and yet survived within the first 28 days of life.

### Operational and term definitions

#### NNM case

It refers to neonates diagnosed with at least one of the near-miss criteria with severe morbidity/clinical criteria or who exhibited pragmatic and/or management criteria but survived this condition within 28 days of their life (25, 26).

#### Antenatal care visits

They were considered to be present if a woman reported having four visits during her current pregnancy (27).

#### Birth weight

It was classified as very low birth weight of <1.5 kg, low birth weight of 1.5–2.5 kg, normal birth weight of 2.5–4 kg, and macrosomia ≥4 kg (26).

#### Term pregnancy

It refers to a woman carrying the pregnancy between 37 completed weeks to 42 completed weeks of gestation (3).

#### Maternal obstetric complication

It refers to mothers who come with one of the following complications: obstructed labor, hypertensive disorders of pregnancy, hemorrhage, sepsis, and other signs indicating complications (27).

### Data analysis procedures

After each questionnaire was checked for completeness and consistency of the information, the data were entered into the EpiData Manager (version 4.4.6) to minimize errors and design skipping patterns. Then, data were exported to a statistical package for social science for cleaning, editing, and analysis purpose. Descriptive analyses (like frequencies, percentages, means, and standard deviation) and summary statistics reflecting the total number of live births and stillbirths were collected from each hospital's records, allowing for the calculation of neonatal outcomes. The neonatal near-miss rate (NNMR) was calculated by dividing the number of neonatal near misses recorded by the number of live births recorded (3, 4, 23).

Assessment of NNM magnitude was limited to the occurrence of one episode of a near-miss event in the first 28 days of newborn life. The analyses of the determinants of neonatal near miss have been restricted to singleton live-born neonates since multiple pregnancies have higher odds of newborn morbidity associated with prematurity and pregnancy complications. We compared categorical variables using X2 square or Fisher's exact test to analyze the risk factor of NNM cases. The variables at a cut-off *p-*value of ≤0.25 on bivariate and variables important from a clinical standpoint were considered for the final model. Statistical significance was declared using a *p-*value < 0.05. Additionally, before running multiple logistic regressions, the variance inflation factor for all predictors and the condition index for model parameters were calculated and checked for collinearity. For all predictors, no problem of collinearity was identified.

Multi-level logistic regression models (three steps) were used to estimate how factors from various levels affect NNM. Outcome variables were entered into the model progressively, from distal to proximal level factors. Three models were constructed, i.e., in Model one, distal level factors were included. Then, subsequent models were constructed by adding covariates at each level of the preceding model. In Model two, intermediate-level factors were added to Model one. The final model, i.e., Model three, was used to estimate the measure of association and was constructed by adding proximal level factors to Model two.

### Data quality assurance

A pre-testing process was conducted on 10% of the total sample size in Canco Hospital. Accordingly, necessary measures were taken to correct the observed error before entering into the actual data collection process. Additionally, data collectors were trained for two consecutive days on the techniques of data collection and the importance of disclosing the aim and purpose of the study to the study participants before the start of data collection. Furthermore, all healthcare workers working in the delivery ward, the postnatal ward, and the neonatal intensive care unit in each participating hospital were also sensitized to the issue. Besides, the inclusion criteria for the neonatal near miss were printed and posted on the walls of each ward at all participating hospitals. The issue of confidentiality throughout the whole process of data collection was discussed and ascertained by the participant. Each questionnaire had the interviewers' initial code to facilitate cross-checking of the completed questionnaire.

## Results

Overall, a total of 7,521 deliveries were conducted in the selected hospital during the study period. After excluding twin delivery (5), stillbirth [63], and non-responding participants (7), a total of 740 live births were included in the study. From the total of 747 mothers with their neonates who took part in the current study, only 740 women completed it, providing a response rate of 99.1%. Of 740 neonates who completed the study, 261 (35.3%) developed a near-miss case.

### Sociodemographic characteristics of the study participant

A total of 747 mothers with their neonates participated in the current study, with 740 women completing the study, providing a response rate of 99.1%. The majority of the women were in the age range of 20–24 years, with a mean age of 27.05 years (SD = 5.6 years). Oromo was the predominant ethnic group including 522 (70.5%) mothers, and 508 (68.6%) of the respondents were orthodox Christianity followers. Of the total respondents, the majority of women, 418 (56.5%) were in marital unions, and 482 (65.1%) resided in urban areas. Of 747 mothers, 509 (68.8%) respondents started a sexual relationship at 18 years of age and 336 (45.4%) women had no job outside the home, and more than one-thirdof the women had no formal education, 264 (35.7%). About one-third of the respondents' partners, 235 (31.8%), were government workers, and 269 respondents (36.4%) did not have any formal education. Table 1 shows the sociodemographic characteristics of the women and their husbands.

**Table 1:** Sociodemographic characteristics of women's neonates admitted to North Shewa zone, public health hospital, Oromia region, Central Ethiopia, 2021.

**Characteristic**	**Category**	**Frequency**	**Percentage**
Age	15–19	48	6.5
	20–24	242	39.2
	25–29	218	29.5
	30–34	141	19.1
	≥35	91	12.3
Ethnicity	Oromo	522	70.5
	Amhara	181	24.5
	Tigre	11	1.5
	Gurage	26	3.5
Religion	Orthodox	508	68.6
	Muslim	103	13.9
	Protestant	111	15.0
	Wakfeta	18	2.4
Women occupation	Government employee	141	19.1
	Merchant	166	22.4
	Daily laborer	97	13.1
	Housewife	336	45.4
Child sex	Male	388	52.4
	Female	352	47.6
Women's education	No formal education	264	35.7
	Primary	162	21.9
	Secondary	197	26.6
	Tertiary and above	117	15.8
Partner's occupation	Government	235	31.8
	Merchant	139	18.1
	Daily laborer	248	33.5
	Farmer	118	15.9
Partner's education	No formal education	269	36.4
	Primary	97	13.1
	Secondary	186	25.1
	Tertiary and above	188	25.4
Marital status	Married	418	56.5
	Cohabiting	188	25.4
	Separated	95	12.7
	Widowed	39	3.8
Relationship status	Living together	606	81.9
with partner	Separated	134	18.1
Residence	Rural	258	34.9
	Urban	482	65.1
Age relationship	<18	231	31.2
started	≥18	509	68.8
With whom	Husband	304	41.1
women living	Husband and child	283	38.2
	Family only	153	20.7
Modes of	Self-referred	461	62.3
hospital admission	Referred from hospital	279	37.7
Means of transport	Ambulance	246	33.2
	Public transport	245	33.1
	Personal vehicles	211	28.5
	Other^*^	38	5.1

* Other, “foot or Animal back”.

### Reproductive health-related characteristics of the respondents

Of the total 740 neonate mothers, 628 (84.9%) respondents had at least one ANC visit during their current pregnancy. However, only 287 (45.7%) respondents had four or more ANC visits. Of the respondents, 557 (75.3%) planned their current pregnancy. Almost half, 358 (48.1%), of the respondents had multipara, 116 (21.4%) had birth order ≥2, and three-fourth, 425 (78.6%), of the mothers had short birth intervals. Regarding delivery modes, 500 (67.7%) respondents gave birth normally through the vagina, and 99 (13.4%) of them had a cesarean section. Of the total neonate mothers, 121 (16.4%) reported a previous history of abortion, with 102 (84.5%) reporting a frequency of one. Regarding the history of stillbirth, about 73 (9.90%) of women had reported a previous history of stillbirth (Table 2).

**Table 2:** Reproductive health-related characteristics of respondents in public hospitals of North Shewa zone, Oromia region, Central Ethiopia, 2021.

**Characteristic**	**Category**	**Frequency**	**Percentage**
Parity	Null parity	199	26.9
	Prim parity	183	24.7
	Multi-parity	358	48.1
Birth interval	Short interval	425	78.6
	Optimal interval	116	21.4
Types of pregnancy	Planned	557	75.3
	Unplanned	183	24.7
ANC follow-up	Yes	628	84.9
	No	112	15.1
Number of ANC	No	112	15.1
follow-up	One	30	4.10
	Two	127	17.2
	Three	184	24.9
	Four	287	38.8
History of abortion	Yes	121	16.4
	No	619	83.6
Frequency of abortion	One	102	84.3
	Two	15	12.3
	Three	4	3.30
History of stillbirth	Yes	73	9.90
	No	667	90.1
Modes of delivery	Normal vagina	500	67.6
	Instrumental	141	19.1
	Cesarean section	99	13.3

ANC, “Antenatal care”.

### Pregnancy-related maternal health characteristics

Of the total 740 respondents who participated in the current study, 67 (9.1%) mothers presented with prenatal bleeding history, while 3.5% of women encountered postpartum hemorrhage. The other potentially life-threatening conditions observed among these women were premature rupture of the membrane, which occurred in 143 (19.3%) respondents, and mal-presentation, in 125 (16.9%) respondents. Approximately 65 (8.8%) women faced pregnancy-related infection, and one-fifth of 143 (19.3%) respondents suffered from premature rupture of the membrane. Around half of newborn mothers, 372 (50.3%), were affected by severe acute malnutrition. Table 3 displays the maternal health-related characteristics.

**Table 3:** Pregnancy-related maternal health characteristics of women in the North Shewa zone, Oromia region, Ethiopia, 2021.

**Variables**	**Category**	**Frequency**	**Percentage**
Prenatal hemorrhage	Yes	67	9.1
	No	673	90.9
Intrapartum hemorrhage	Yes	36	4.9
	No	704	95.1
Postpartum hemorrhage	Yes	26	3.5
	No	714	96.5
Preeclampsia	Yes	68	9.2
	No	672	90.8
Pregnancy-related infection	Yes	65	8.8
	No	675	91.2
Mal presentation	Yes	125	16.9
	No	615	83.1
Obstructed labor	Yes	49	6.6
	No	691	93.4
Premature rupture	Yes	143	19.3
of membrane	No	597	80.7
Hypotension	Yes	15	2.00
	No	725	98.0
Uterine prolapse	Yes	20	2.70
	No	720	97.3
Placenta abruption	Yes	14	1.90
	No	726	98.1
MUAC of mother	SAM	372	50.3
	No malnutrition	368	49.7

MUAC, Mid-upper arm circumference; SAM, Severe Acute Malnutrition.

### Child health-related characteristics

Of the total newborns delivered in a selected hospital, 165 (22.3%) neonates required emergency lifesaving treatment and were admitted to the neonatal intensive-care unit (NICU). Of the total 165 (22.5%) neonates admitted to the NICU center, 31 (18.7%) neonates stayed <2 days, 108 (65.5%) neonates stayed for 2–6 days, 15 (9.0%) neonates stayed for 7–11 days, and 11 (6.7%) neonates stayed for more than 12 days. With respect to birth asphyxia at the fifth minute of delivery, 25 (3.4%) neonates had severe asphyxia, whereas 116 (15.7%) had moderate birth asphyxia. Regarding body temperature at birth, 38 (5.1%) newborns were hypothermic. Regarding birth weight, 17 (2.3%) had very low birth weights (Table 4).

**Table 4:** Child health-related characteristics among neonates delivered in North Shewa Zone Public Health Hospital, Oromia region, Central Ethiopia, 2021.

**Variables**	**Category**	**Frequency**	**Percentage**
1st minute APGAR	Severe asphyxia	49	6.60
	Moderate asphyxia	267	36.1
	No asphyxia	424	57.3
5th minute APGAR	Severe asphyxia	25	3.40
	Moderate asphyxia	116	15.7
	No asphyxia	599	80.9
7th minute APGAR	Severe asphyxia	19	2.60
	Mild asphyxia	65	8.80
	No asphyxia	656	88.6
Birth weight	VLBW	17	2.30
	LBW	150	20.3
	Normal	573	77.4
Body temperature	Hypothermic	38	5.10
	Normal	702	94.9
Admission to NICU	Yes	165	22.3
	No	575	77.7
Length of NICU stay	<2 days	31	18.7
	2–6 days	108	65.5
	7–11 days	15	9.0
	≥12 days	11	6.7

APGAR, Appearance, Pulse, Grimace, Activity, and Respiratory; NICU, Neonatal Intensive Care Unit; VLBW, Very Low Birth weight; LBW, Low Birth Weight.

### NNM identification criteria used in the north zone

An APGAR score at the fifth minute was the most common pragmatic criteria for identifying neonatal near-miss cases, which was recorded in 141 neonates (52.8%), and it corresponded to a prevalence of 19.1 per 1000 live births. The absence of regular breathing 60 (30%) and the inability to suckle within 12 h 56 min (28%) was the most common clinical criterion used in identifying the NNM case, accounting for almost 8.1% and 7.6%/1,000 live births, respectively. Among the management criteria of NNM selection criteria, parenteral antibiotic was the most commonly identified case in 95 (44.8%) respondents, with a proportion of 12.8/1,000 live births, and 78 (36.8%) cases of neonatal near miss represented intubation, with a proportion of 10.5/1,000 live births (Table 5).

**Table 5:** Criteria used for the identification of neonatal near miss, North Shewa zone, Oromia region, Ethiopia, 2021.

**Criteria**	**Frequency NNM (n)**	**Prevalence of NNM (%) per 1,000 live birth**
**Pragmatic criteria**		
Gestational age ≤33wks	109 (40.8)	14.7
APGAR score <7 at 5^th^ min	141 (52.8)	19.1
Birth weight (gram) < 1500g	17 (6.4)	2.3
**Clinical criteria**		
Cyanosis	44 (22.0)	5.9
Absence of regular breathing	60 (30.0)	8.1
Cardiac arrest	20 (10.0)	2.7
Persistent bradycardia < 80 bpm	8 (4.0)	1.1
Visible jaundice within 24 h of delivery	—	—-
Persistent tachycardia > 200bpm	8 (4.0)	1.1
Seizures	—	—
Inability to suck within 12 hr	56 (28.0)	7.6
Anuria > 24	—	—
Tachypnea	4 (2.0)	0.5
**Laboratory and management criteria**		
Any active non-traumatic bleeding	—	—
Hematuria	2 (0.9)	0.3
Septicemia	—	—
Encephalopathy	—	—
Any intubation	78 (36.8)	10.5
Cardiopulmonary resuscitation	30 (14.2)	3.9
Use of vasoactive drugs	4 (1.90)	0.5
Blood transfusion	3 (1.40)	0.4
Anticonvulsant	—-	—
Parenteral antibiotic	95 (44.8)	12.8

Apgar (A, Appearance; P, Pulse; G, Grimace; A, Activity; R, Respiration); bpm, beat per minute; g, gram; h, hours; wks, weeks.

### The magnitude of neonatal near miss

**Figure 3:**
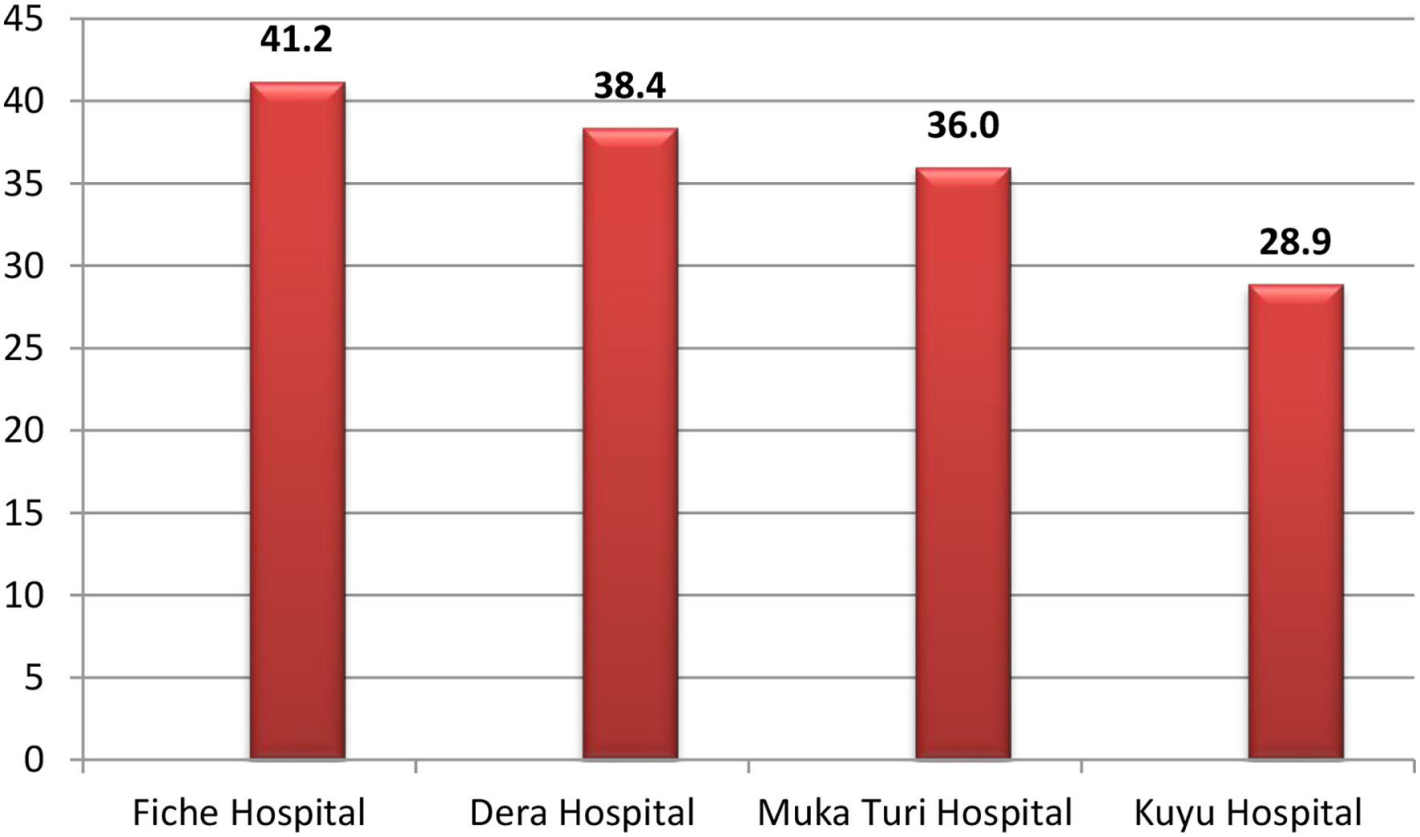
Percentage distribution of near-miss cases among newborns delivered in each public health hospital of North Shewa zone, Central Ethiopia, 2021.

The overall magnitude of the neonatal near-miss rate was 35.3% (95% CI = 31.9–38.6). Higher near-miss cases were reported from Fiche hospital with 100 (41.2%) cases, followed by 50 (36.0%) cases from Muka Turi hospital. Thirty-three (28.9%) cases were reported from Kuyu hospital (Figure 3). The magnitudes of NNM were high both at the early age of newborns, i.e., 52.1% within the first 24 h of delivery and 33.0% in the early neonatal period. Only 14.9% of the newly delivered babies in the late neonatal period developed near-miss cases.

### Factor associated with neonatal near miss

We attempted to identify risk factors, i.e., those associated with a near neonatal miss, using bivariate logistic regression with the analysis of model fit statistics. Model I for distal factors (participant occupation, age at marriage, marital status, modes of hospital admission, and current status to their partner) showed an association on bivariate logistic regression. After adjusting for all distal level factors, only participant occupation and marital status showed a statistical association. In model II, the respondent's occupation, marital status, and history of previous abortion showed a statistically significant association with a neonatal near miss. After adding proximal factors in model III (controlling for all variables), participant occupation [AOR: 0.55, CI: 0.33–0.90], marital status [AOR: 2.19; CI: 1.06–4.51], instrumental delivery [AOR: 1.98; CI: 1.10–3.55], intrapartum hemorrhage [AOR: 2.27; CI: 1.03–5.01], abortion history [AOR: 1.59; CI: 1.03–2.44], mal-presentation [AOR: 1.77; CI: 1.14–2.77], premature rupture of membrane [AOR: 2.36; CI: 1.59–3.51], and pregnancy-related infection [AOR: 1.99; CI: 1.14–3.46] were significantly associated with near-miss cases of neonates (Table 6).

**Table 6:** Determinant of NNM among neonates delivered in North Shewa zone public health facility, Oromia region, Ethiopia, 2021.

**Variables**	**Neonatal near miss**		**COR (95%CI)**	**Mode-I** **AOR (95%CI)**	**Model-II** **AOR** **(95%CI)**	**Model-III** **AOR (95%CI)**
	**Yes**	**No**				
**Distal factors**						
**Respondent occupation**						
Government	56 (39.7)	85 (60.3)	1	1	1	1
Merchant	82 (31.4)	82 (49.4)	1.48 (0.94–2.33)	1.50 (0.94–2.39)	1.47 (0.88–2.47)	1.51 (0.88–2.60)
Daily laborer	32 (33.0)	65 (67.0)	0.74 (0.43–1.28)	0.61 (0.32–1.15)	0.61 (0.32–1.15)	0.57 (0.29–1.13)
Housewife	91 (27.1)	245 (72.9)	0.56 (0.37–0.85)	0.59 (0.37–0.94)	0.59 (0.37–0.94)	0.55 (0.33–0.90)^*^
**Age at marriage**						
< 18yrs	72 (31.2)	159 (68.8)	1.30 (0.93–1.81)	1.16 (0.82–1.65)		
≥18yrs	189 (37.1)	320 (62.9)	1	1		
**Marital status**						
Married	127 (30.4)	291 (69.6)	1	1		
Cohabiting	68 (36.2)	120 (63.8)	1.29 (0.89 −1.86)	1.14 (0.77–1.67)	1.21 (0.83–1.76)	1.22 (0.82–1.82)
Separated	45 (47.4)	50 (52.6)	2.06 (1.31 −3.24)	1.69 (1.01–2.81)	1.97 (1.23–3.16)	1.84 (1.12–3.01)^*^
Widowed	21 (53.8)	18 (46.2)	2.62 (1.37–5.18)	1.84 (0.88–3.83)	2.07 (1.04–4.12)	2.19 (1.06–4.51)^*^
**Modes of admission**						
Self-referred	154 (33.4)	307 (66.6)	1.24 (0.91 −1.69)	1.22 (0.89–1.68)		
Referred from other facilities	107 (38.4)	172 (61.6)	1	1		
**Relationship status with current**						
**Partner**						
Living together	196 (32.3)	410 (67.7)	1.97 (1.34–2.87)	1.48 (0.94–2.32)		
Separated	65 (48.5)	69 (51.5)	1	1		
**Intermediate factors**						
**Types of pregnancy**						
Planned	190 (34.1)	367 (65.9)	1		1	
Unplanned	71 (38.8)	112 (61.2)	1.22 (0.86–1.74)		1.18 (0.82–1.70)	
**History of abortion**						
Yes	57 (47.1)	64 (52.9)	1.81 (1.22–2.68)		1.69 (1.12–2.55)	1.59 (1.03–2.44)^*^
No	204 (33.0)	415 (67.0)	1		1	1
**History stillbirth**						
Yes	32 (43.8)	41 (56.2)	1.49 (0.91–2.43)		1.54 (0.92–2.57)	
No	229 (34.3)	438 (65.7)	1		1	
**Proximal factor**						
**Mode of delivery**						
Spontaneous	157 (31.4)	343 (68.6)	1			1
Instrumental	66 (46.8)	75 (53.2)	1.92 (1.31–2.81)			1.98 (1.10–3.55)^*^
Cesarean section	38 (38.4)	61 (61.6)	1.36 (0.87–2.12)			0.95 (0.58–1.55)
**Current prenatal bleeding**						
Yes	34 (50.7)	33 (49.3)	2.02 (1.22–3.35)			1.27 (0.69–2.34)
No	227 (33.7)	446 (66.3)	1			1
**Intrapartum hemorrhage**						
Yes	19 (52.8)	17 (47.2)	2.13 (1.08–4.18)			2.27 (1.03-5.01)^*^
No	242 (34.4)	462 (65.6)	1			1
**Preeclampsia**						
Yes	33 (48.5)	35 (51.5)	1.83 (1.11–3.03)			1.44 (0.83–2.49)
No	228 (33.9)	444 (66.1)	1			1
**Pregnancy-related infection**						
Yes	35 (53.8)	30 (46.2)	2.31 (1.38–3.87)			1.99 (1.14–3.46)^*^
No	226 (33.5)	449 (66.5)	1			1
**Mal-presentation**						
Yes	56 (44.8)	69 (55.2)	1.62 (1.09–2.39)			1.77 (1.14–2.77)^*^
No	205 (33.3)	410 (66.7)	1			1
**PROM**						
Yes	72 (50.3)	71 (49.7)	2.18 (1.51–3.17)			2.36 (1.59–3.51)^*^
No	189 (31.7)	408 (68.3)	1			1
**Placenta abruption**						
Yes	9 (64.3)	5 (35.7)	3.38 (1.12-10.21)			2.95 (0.88–9.88)
No	252 (34.7)	456 (65.3)	1			1
Test of Model fitness				Model I	Model II	Model III
Likelihood ratio			–	−916.882	−911.0.064	−862.363
AIC			–	1,761	1,534	1,267
VIF				1.351	1.026	1.062
Adjusted R square				0.063	0.042	0.083

VIF, Variance inflation Factors; AIC, Akaika information criterion; PROM, Premature rupture of membrane; ANC, Anti-natal care; COR, Crude odds ratio; AOR, adjusted odds ratio;

* Significant variables.

## Discussion

Evidence of near neonatal miss is an essential tool in assessing the quality of care provided to neonates, and several factors can contribute to a near miss. The study's results contributed to the understanding of the magnitude and factors associated with life-threatening conditions in neonates. The magnitude of the near-miss cases was 35.3%, with Fiche Hospital reporting higher cases (41.2%). A significant proportion (52.1%) of near-miss cases occurred within the first 24 h after delivery.

The proportion of neonatal near misses (35.3%) in the current study was comparable with the finding from Uganda in 2014 (28). A possible explanation might be that both studies used analogous measuring tools. However, the current study's findings were lower than those of a study conducted in the Southern Nation Nationalities and Peoples' Region, Ethiopia in (2018), which showed that 45.1% of neonates were nearly missed (21). A possible explanation for the difference in the results may be that the study conducted in the southern part used a larger sample size compared to the current study, providing an adequate chance of eliciting cases. Additionally our finding was lower than the findings of the study conducted among neonates in Brazil (24). This discrepancy might be due to variations in the health conditions of the women included, whereas the previous study included only women with potentially life-threatening conditions, which may result in an increased chance of case occurrence.

Furthermore, variation in the occurrence of near-miss cases was observed within hospitals, which was 41.2, 36.0, 34.08, and 28.9% from Fiche, Mukaturi, Dera, and Kuyu hospitals, respectively. A higher proportion of near-miss cases were reported from general hospitals than from other district hospitals. The variation may be because general hospitals use more sophisticated diagnostic and management protocols than most hospitals. This might provide a better chance to adequately elicit near-miss case occurrences.

The studies found various predictors of neonatal near-miss cases among newly delivered babies. It demonstrated that marital status, having an abortion history, and having a pregnancy-related infection, intrapartum hemorrhage, participant occupation, modes of delivery were identified as predictors. Additionally, conditions indicating severe potential life-threatening conditions, such as mal-presentation and premature rupture of the membrane, were also identified as determinants of a neonatal near miss. Newborns born to widowed mothers were more likely to be at risk of developing life-threatening conditions than those born to married women. Similar findings were reported from a study conducted in Brazil (24). There is evidence that infants of pregnant mothers whose partners died are more likely to have behavioral issues and engage in dangerous behaviors. The absence of a partner was associated with a higher prevalence of depression, stress, and anxiety during pregnancy (29), increased consumption of illicit drugs (30), and inadequate prenatal care (31), all of which are factors that may result in lower birth weight of the child, preterm birth, or congenital malformations, which would be indicators of NNM.

Neonates from mothers with a history of abortion were more likely to develop severe illness. Similarly, the study conducted in the Gurage zone (in 2020), Guji, and Borana zones support the finding that abortion history predisposes the child to a life-threatening condition (32, 33). A possible justification might be that women with a previous history of abortion may suffer short- and long-term consequences such as infection, cervical laceration, uterine atony, and perforation (34). This might result in an increased risk of subsequent premature birth, low birth weight, low APGAR score, and intra-uterine growth retardation (35). This might show the need to decrease the incidence of abortion by strengthening family planning services and improving women's sexual and reproductive health knowledge, which helps to mitigate the adverse effects of abortion.

There is substantial evidence of the close association between current maternal and infant health issues. The main maternal factors identified in this study imply that the more complicated the mother's clinical condition, the worse her perinatal outcome. Any infection that occurs during pregnancy or immediately after childbirth can jeopardize the baby's life (36). Thus, preventing or diagnosing and treating infections during pregnancy appropriately and timely can potentially save both the mother and the baby from developing a severe neonatal condition (37).

Although there are limited similar studies to compare with our result on the association between instrumental delivery and neonatal near miss, one study conducted in Ambo, Ethiopia showed an association between a near miss and instrumental delivery (38). This may be because instrumental delivery has adverse effects such as skull fracture and/or intracranial hemorrhage, fetal acidosis, and cervical spine injury, which may lead newborn babies to develop severe life-threatening conditions (39, 40). Improving the proficiency of healthcare professionals to reduce the adverse effects of instrumental delivery is mandatory.

Obstetric complications during the late antepartum period are one of the main factors associated with a potentially life-threatening condition in neonates (41). In this study, preterm premature rupture of the membrane was strongly associated with a neonatal near miss. This finding is consistent with the study conducted at Ingibara, Debre Tabor, and Gamo Gofa (42–44), showing that premature rupture of the membrane increases the odds of an NNM case by two times as compared to their counterpart. The occurrence of severe neonatal conditions in the case of preterm rupturing of the membrane may be due to those stated conditions. In one way or another, it can affect the neonates during intra-uterine and extra-uterine life and predispose them to life-threatening conditions such as sepsis, pulmonary hypoplasia, chorioamnionitis, and respiratory distress syndrome (44–46). Ascertaining individual-level risk factors, followed by an appropriate treatment plan, was crucial in reversing the problem.

Abnormal bleeding during labor was identified as the determinant of neonatal near misses. This finding is congruent with the study conducted in Ethiopia's southern and eastern parts (44, 47). The possible explanation may be that intrapartum hemorrhage may be related to placenta abruption, placenta praevia, and possibly uterine rupture, which is often a life-threatening obstetric emergency for the fetus associated with hypoxia, cyanosis, and other problems, which may increase the chance of near-miss occurrence (48). Early detection and close monitoring during intrapartum care are crucial to reducing adverse fetal outcomes.

There is published evidence for the adverse effects of non-vertex presentation and neonatal conditions (44, 49). The current study also revealed that the odds of an NNM case were two times higher among neonates with non-vertex presentations than those with a normal presentation. This might be because of mal-presentation during pregnancy, and women in labor with this condition experience a high risk of fetal distress, uterine rupture, and other complications and may also lead to obstructed and prolonged labor, which may result in a severe life-threatening condition for the newborn (50, 51).

In this study, the mother's employment status was significantly associated with the occurrence of near-miss incidents. Newborns of unemployed mothers were at an increased risk of near miss compared with those employed. Unemployment can be expected to affect fetal development due to stress, resulting from reduced financial wellbeing, perceived pressure to find employment, changes in daily routine, and self-perception that follow job loss (1, 52). However, the impact of unemployment on newborn health could be mitigated by less purchasing power (53). In contrast to what we observed, Anteneh et al. (54) reported that the offspring of employed mothers were at a higher risk of near miss than those of non-employed mothers. This difference should be the subject of a qualitative study and a robust study plan.

### Limitations of the study

The notable strength of this study's near-miss case was broadly defined, i.e., three criteria (pragmatic, clinical, and management) were used. These prevent an underestimation of the proportion of near-miss cases in the population. In addition, newborns were monitored throughout the neonatal period (from birth to 28 days of life), which minimizes the risk of under-reporting of near-miss cases. The study, however, has some limitations as some management variables that help to characterize an NNM case, such as phototherapy and surgery, were absent, and this may lead to an underestimation of the prevalence of NNM as none of the selected hospitals were providing phototherapy and surgical care for the newborns. Furthermore, the study did not consider multiple episodes of NNM events that occurred in a neonate. Additionally, newborns referred from other health facilities and delivered at home were excluded from the study, as it was difficult to obtain information on the condition of newborns at birth, such as APGAR score and birth weight, possibly resulting in the underestimation of near-miss cases.

## Conclusion

The study notes that the extent of NNM in newborns is a major public health concern. Factors acting at proximal and intermediate levels showed a more significant effect on neonatal near misses compared to factors acting at distal levels. Modifiable obstetric risk factors, such as abortion history, pregnancy-related infection, intrapartum hemorrhage, premature rupture of the membrane, and mal-presentation, have shown significant association with a neonatal near miss. As a result, addressing modifiable obstetric risk factors through the provision of skilled and quality care to mothers during pregnancy, as well as during and after childbirth, was important for improving neonatal health. Additionally, strengthening antenatal care services to minimize infection occurring during pregnancy through the provision of appropriate services and counseling about the consequences of abortion was essential in reversing the problem.

## Data availability statement

The original contributions presented in the study are included in the article/supplementary material, further inquiries can be directed to the corresponding author/s.

## Ethics statement

Ethical approval was obtained from Salale University Ethics Review Committee. Written informed consent from the patients/participants was not required to participate in this study in accordance with the national legislation and the institutional requirements. Informed verbal consent was obtained from the mothers of newborns before conducting the interviews.

## Author contributions

GG generated the idea, developed study protocols, supervised data collection, entered the data, conducted analysis, interpreted the data, and drafted the final manuscript. BD, MW, and SD assisted with the study protocol development, data interpretation, and reviewed the manuscript critically. AA assisted with the study protocol development, data interpretation, reviewed the manuscript critically, and drafting the study. All authors contributed to the article and approved the submitted version.

## Funding

This study was funded by Salale University, Health Science College, and the Public Health Department (SLU882-21-13).

## Conflict of interest

The authors declare that the research was conducted in the absence of any commercial or financial relationships that could be construed as a potential conflict of interest.

## Publisher's note

All claims expressed in this article are solely those of the authors and do not necessarily represent those of their affiliated organizations, or those of the publisher, the editors and the reviewers. Any product that may be evaluated in this article, or claim that may be made by its manufacturer, is not guaranteed or endorsed by the publisher.

## Abbreviations

NNM: neonatal near miss
SDG: sustainable development goal
WHO: World Health Organization
ANC: antenatal care
AOR: adjusted odds ratio
COR: crude odds ratio
NICU: neonatal intensive care unit
SD: standard deviation
CS: cesarean section.
